# Nd:YVO_4_ Laser Irradiation on Cr_3_C_2_-25(Ni20Cr) Coating Realized with High Velocity Oxy-Fuel Technology—Analysis of Surface Modification

**DOI:** 10.3390/mi12121477

**Published:** 2021-11-29

**Authors:** Luca Giorleo, Giovina Marina La Vecchia, Elisabetta Ceretti

**Affiliations:** Department of Mechanical and Industrial Engineering, University of Brescia, 25123 Brescia, Italy; marina.lavecchia@unibs.it (G.M.L.V.); elisabetta.ceretti@unibs.it (E.C.)

**Keywords:** laser surface modification, HVOF coating, tailored surfaces

## Abstract

The high-velocity oxy-fuel (HVOF) technique has been extensively used for the deposition of hard metal coatings. The main advantage of HVOF, compared to other thermal spray techniques, is its ability to accelerate the melted powder particles of the feedstock material to a relatively high velocity, leading to good adhesion and low porosity. To further improve the surface properties, a mechanical machining process is often needed; however, a key problem is that the high hardness of the coating makes the polishing process expensive (in terms of time and tool wear). Another approach to achieving surface modification is through interaction with a thermal source, such as a laser beam. In this research, the effects of laser scanning rate, scanning strategy, and number of loop cycles were investigated on an HVOF-coated surface. Cr_3_C_2_-25(Ni20Cr) was selected as the coating and Nd:YVO_4_ as the laser source. The results demonstrate the significance of the starting coating morphology and how the laser process parameters can be tuned to generate different types of modifications, ranging from polishing to texturing.

## 1. Introduction

There exists an increasing need to reduce and control friction and wear, in order to extend the lifetime of mechanical systems to improve their efficiency and reliability; in particular, surface modifications play a fundamental role in enhancing the performance of mechanical parts. To improve surface part properties, at present, two solutions are mainly applied: modifying the surface morphology with laser irradiation or enhancing the mechanical properties using a surface coating.

When considering the use of lasers, the modification of surface properties plays an important role in optimizing a material’s performance for a given application. The unique interaction of laser light with a material can lead to permanent changes in the material’s properties, which is not easily achievable through other means. Laser irradiation has been shown to induce changes in local chemistry, local crystal structure, and local morphology, all of which affect how a material behaves in a given application [1]. The effect of heating a material with laser radiation depends on the thermal balance of the surface-absorbed laser energy and the thermal energy transferred to the material. When the rate of laser energy deposition is much higher than the rate of heat transfers to the core material, the high-temperature zone is localized in a thin surface layer [2,3]. Laser beams, due to their high coherence and directionality, have been widely used for the surface modification of many kinds of metals [4] and, depending on the laser process parameters, it is possible to induce different morphology modifications, ranging from polishing to texturing, on the treated surface. Laser polishing is a finishing process which mainly includes the melting of a thin layer of a metal’s surface without any cracks or surface defects [5]. This process aims to smooth the peaks that are found on the metal surface to an intermediate range below the peaks [6]. This process has found potential application in reducing the surface roughness of parts, which has been realized through the use of additive manufacturing technology [7]. To the contrary, surface texturing has emerged in the last decade as a viable option for surface engineering, resulting in significant improvements in load capacity, wear resistance, and the friction coefficient of mechanical components [8,9,10]. Generally, this technique induces micro-dimples [11], dimple arrays [12], or micro grids [13] on surfaces, improving not only the described mechanical properties but also, in part, the associated lubrication. The main limits of laser surface modification are due to the bulk material; indeed, the improvement that is possible to achieve is strictly limited by the properties of the treated material.

Coatings are often used to resist severe wear in diverse industrial applications. Several applications are available for various coating thicknesses, from macro- to nano-scale [14]. For macro-coatings, the evolution of high velocity oxy-fuel (HVOF) deposition processes has provided denser and more homogeneous coatings with less carbide decomposition than those from other spray processes, mainly due to the combination of higher kinetic energy and lower spray temperatures [15,16]. HVOF coatings have found applications in various industrial applications, including mining, mineral or pulp and paper processing, aerospace and automobile manufacturing, and power generation [17]. However, despite these benefits, the coating deposition process is typically characterized by a lower surface quality in terms of roughness and a mechanical finishing process is often required. The finishing process, as a result, can become very expensive due to the required properties of the coating.

To achieve the benefits of both techniques, hybrid processes are now available: coatings are irradiated by a laser source to induce a polishing, cleaning, or texturing process. The benefits achieved by such a combination include increased corrosion resistance [18], roughness reduction [19,20], and/or enhanced wear properties [21].

To enhance knowledge regarding this topic, in this article, we investigate the effect of laser irradiation on a Cr_3_C_2_-25(Ni20Cr) coating realized with HVOF. Cr_3_C_2_-25 coatings have been widely used in high-temperature (850 °C) industrial applications due to their structural stability and retention of mechanical properties at higher service temperatures [22]. A variety of techniques can be used to prepare chromium carbide-based coatings, among which HVOF processes have been widely used, as they produce smooth, low-porosity, dense, and adherent coatings, without significantly altering the integrity of the carbide particles [23]. Some preliminary studies presented in the literature that focus on laser surface modification of carbide coatings are detailed in the following: Morimoto et al. tested the effect of a diode laser on a cermet coating, achieving an increase in surface hardness [24]; Sun et al., using a fiber laser, tested the effect of scan speed on wear behavior and observed that the wear resistance of the optimized layer was increased by 29.76 times [25]. Scendo et al. investigated the influence of the CO_2_ laser remelting process, and found that the micro-hardness of a cermet coating decreased as the speed of laser irradiation increased [26]; Goral et al., presented a study considering the effect of laser surface treatment on microstructure and mechanical characteristics, highlighting that cermet coatings were characterized by compressive residual stresses whose value increased with higher laser power [27]. The main contribution of this research with respect to the cited literature is that we analyze the effect of process parameters, including the laser scanning rate, scanning strategy, and loop cycle of an untested laser source (Nd:YVO_4_), in order to measure the effect of laser surface modification and, thus, analyze the potential of this laser source to treat cermet carbide.

## 2. Materials and Methods

For this research, three different experimental series were designed to analyze the interaction between the laser source and the HVOF-treated sample. In the first and second series, the effects of five levels of laser scanning rate and three irradiation strategies were tested, respectively, on the HVOF sample (considering as-sprayed and after shot peening conditions). In the third experimental series, the effect of laser repetition cycles (loops) was investigated on the HVOF sample (considering after shot peening conditions), analyzing the effects of four different levels of looping.

**Figure 1:**
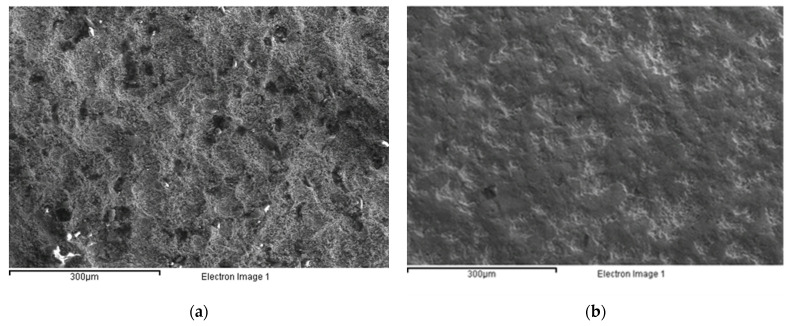
Morphology of sample after HVOF coating (**a**); and after HVOF and shot peening (**b**).

HVOF-processed carbon steel SAE 1070 samples with dimensions of 76 × 19 × 2 mm^3^ were selected as substrate, and Cr_3_C_2_-25(Ni20Cr) with 80% Cr_3_C_2_ (% weight) was used as the powder. The powder has globular geometry with an average grain size between 11 and 45 µm. The powder was sprayed using the HVOF technique with standard spraying parameters: 850 nL/h of oxygen, 450 mL/min of kerosene, a powder feed rate of 70 g/min, and a spray distance of 355 mm. The coating had a hardness of 700–800 HV0.3, a porosity of 1.5%, a maximum operating temperature of around 800 °C, and a high resistance to anchoring with compression above 80 MPa. Figure 1 shows the morphology of the samples produced after HVOF treatment (Figure 1a) and after shot peening (Figure 1b). The coating thickness was measured as equal to 200 µm.

**Figure 2:**
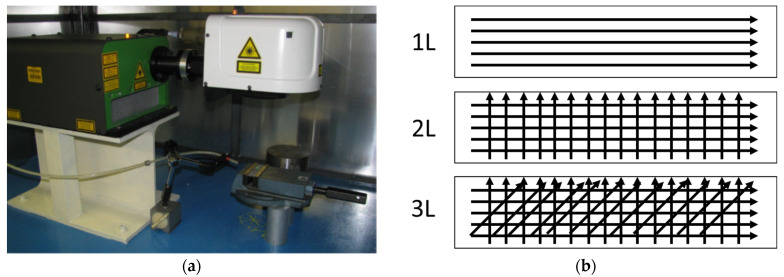
Laser micro-machining layout setup (**a**) and strategy parameters (**b**).

Laser micro-machining was performed on the coated samples using an Nd:YVO_4_ laser (Lee Laser LEP-V20MGQ). The outgoing laser beam was collimated in a galvanometer scanner system, in order to impose remote control. The laser pattern (or filling strategy) was designed using the Laser Marking Studio (LMS) software integrated into the galvanometer scanner system. Figure 2a reports the setup of the experiment.

In the first and second series, the effects of five levels of laser scanning rate and three irradiation strategies were tested, respectively, on as-sprayed and after shot peening HVOF samples. The five levels of laser scanning rate tested ranged between 100 and 300 mm/s, with a step increment of 50 mm/s. With respect to the filling strategy, the aim was to investigate a solution that generates a tailored surface along the x direction (i.e., one-line strategy, 1 L), along the x and y directions (i.e., two-line strategy, 2 L), and along the x and y directions as well as a direction at 45° with respect to the galvanometric system axis (i.e., three-line strategy, 3 L). For the object of the third series (loops), four levels were fixed: 1, 5, 10, or 20 loops. Figure 2b describes the laser single scan as a function of the scan strategy, while Table 1 and Table 2 report the fixed and variable laser process parameters, respectively.

Qualitative and quantitative analyses of the structures obtained in all the experimental series were performed. To obtain qualitative information about the laser irradiation, we analyzed the sample surface morphology using a scanning electron microscope (LEO EVO 40). For the quantitative analysis, a laser probe system (Mitaka PF60) was used to acquire the arithmetic mean of ordinates of the roughness profile (Ra) and the average maximum peak-to-valley height of the profile (Rz). For each experimental series, five measurements were acquired along the x and y directions. The roughness measures were acquired according to ISO 4287. The results were analyzed using an analysis of variance (AnoVa), in order to estimate the statistical significance of the tested parameters. In particular, different comparison methods were imposed as a function of the starting surface roughness of the samples.

Finally, in the third experimental series, to better analyze the surface morphology obtained when increasing the number of loop cycles, a quantitative analysis was performed using a 3D digital microscope [(Hirox RH-2000)] and Tukey’s range test (so-called reasonable important difference), in order to find means that were significantly different from each other.

## 3. Results

### 3.1. Preliminary Experimental Series

**Figure 3:**
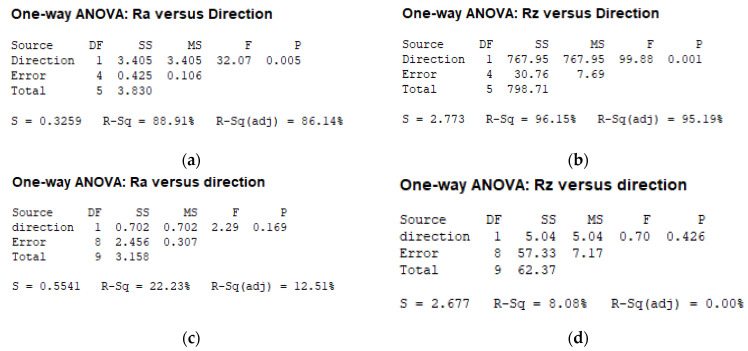
AnoVA analysis of x and y directions vs. the initial average surface roughness (Ra) and average peak-to-valley height (Rz) for as-sprayed (**a**,**b**) and after shot peening (**c**,**d**) HVOF-treated samples.

Figure 3 reports the AnoVa results of a preliminary series, executed on as-sprayed and shot peened samples before laser irradiation. As described in Figure 3a,b, the as-sprayed sample results significantly differed in terms of Ra and Rz along the x and y directions (*p* < 0.005); to the contrary, Figure 3c,d report how the direction became non-significant after the shot peening treatment (*p* > 0.005). Based on this preliminary analysis, in the first experimental series we report the analysis results as a function of the direction, while in the second and third experimental series we do not. Table 3 reports the mean and average Ra and Rz values of the as-sprayed and after shot peening results.

### 3.2. First Experimental Series

**Figure 4:**
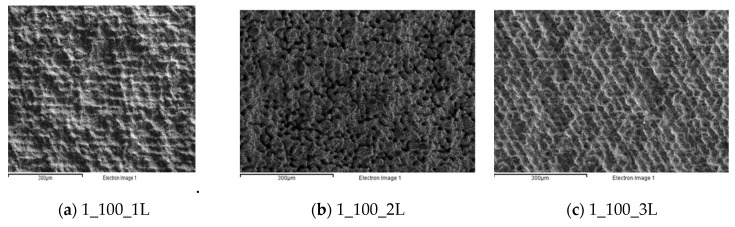

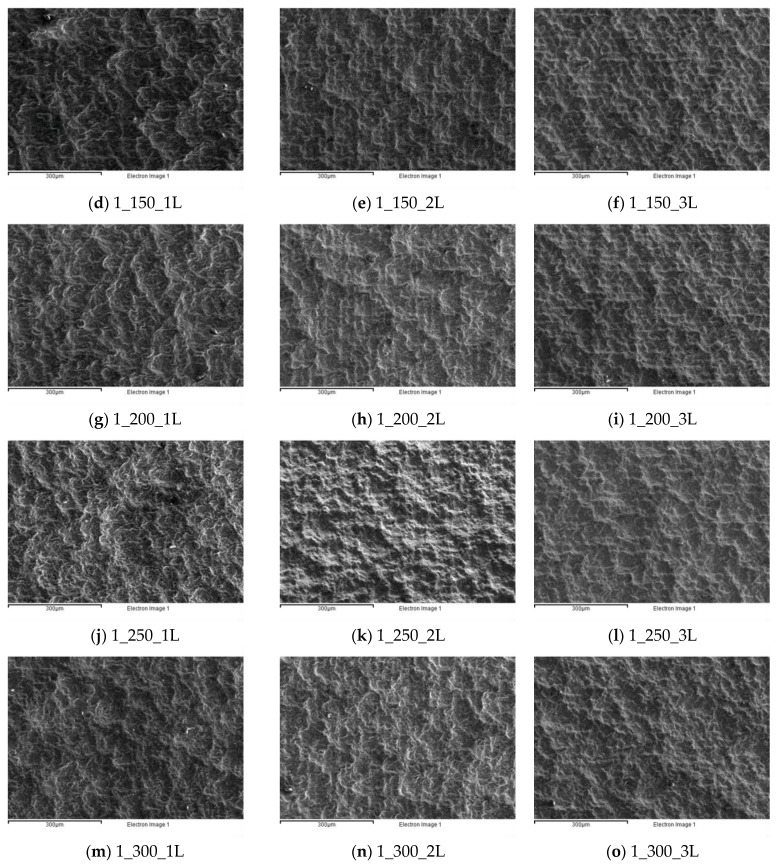
Morphological analysis for the first experimental series. (**a**) 1_100_1L; (**b**) 1_100_2L; (**c**) 1_100_3L; (**d**) 1_150_1L; (**e**) 1_150_2L; (**f**) 1_150_3L; (**g**) 1_200_1L; (**h**) 1_200_2L; (**i**) 1_200_3L; (**j**) 1_250_1L; (**k**) 1_250_2L; (**l**) 1_250_3L; (**m**) 1_300_1L; (**n**) 1_300_2L; (**o**) 1_300_3L.

**Figure 5:**
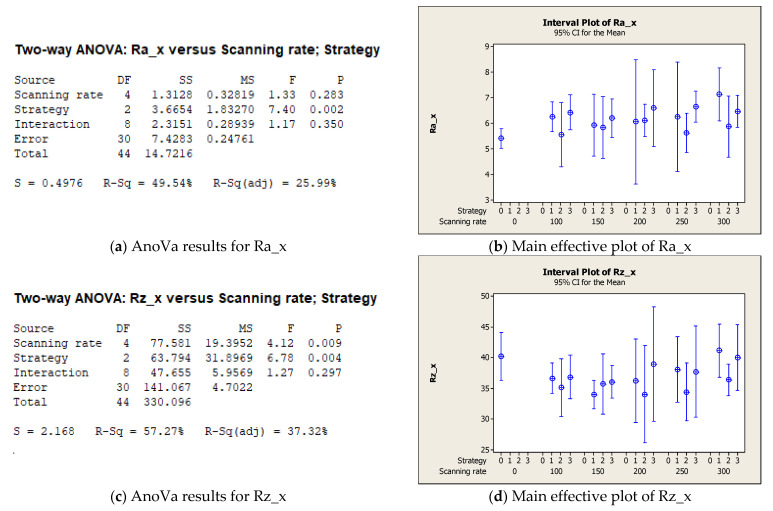
Main results of statistical analysis along the x direction. (**a**) AnoVa results for Ra_x; (**b**) Main effective plot of Ra_x; (**c**) AnoVa results for Rz_x; (**d**) Main effective plot of Rz_x.

**Figure 6:**
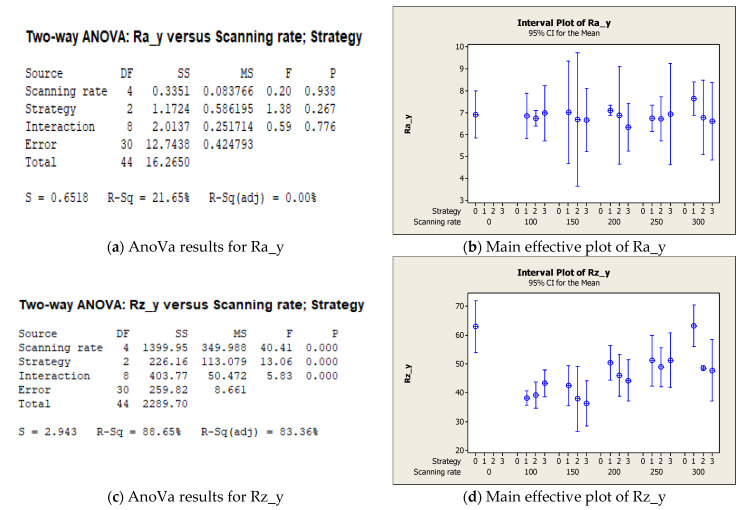
Main results of statistical analysis along the y direction. (**a**) AnoVa results for Ra_y; (**b**) Main effective plot of Ra_y; (**c**) AnoVa results for Rz_y; (**d**) Main effective plot of Rz_y.

The main results referring to the first experimental series are reported in terms of surface morphology (Figure 4) and statistical analysis (Figure 5 and Figure 6). The coding adopted to identify the samples was to name each test in the following format: (experimental series)_(laser scanning rate)_(laser strategy).

Figure 4 highlights that, from a quantitative point of view, the single-line strategy (1_L) was able to generate a pattern on the substrate only at a low scanning rate (see Figure 4a,d). To the contrary, when the laser scanning rate increased, tailored surfaces were generated in the case of the two- or three-line strategies (see Figure 4k,l). Moreover, compared with the initial morphology shown in Figure 1a, a smoother surface can be seen.

Regarding the statistical analysis, different considerations could be deduced as a function of the direction. For the x direction analysis, Figure 5 reports that not only was the effect of laser scanning rate non-significant for Ra and Rz (see Figure 5a,d) but, to the contrary, it is possible to see from Figure 5b,d that the data dispersion increased due to laser irradiation (as-sprayed Ra_x and Rz_x are also reported in the beginning of the graphs, coded as 0). Regarding the laser strategy, as observed above, a significant effect emerged from the AnoVa, for the two-line strategy in particular.

Regarding the analysis along the y direction of the as-sprayed sample, Figure 6 confirms that the average roughness, Ra, was not affected by the tested process parameters (see Figure 6a,b); however, on the surface, as observed in the morphological analysis, a significant decrease in Rz was achieved in particular when the laser scanning rate was equal to 100 or 150 mm/s (see Figure 6d). The laser strategy still produced significant results for Rz (Figure 6c) and, as confirmed by Figure 6d, good results were achieved with the two-line strategy. In the main effective plot, the value (coded as 0) of the as-sprayed sample is also reported, confirming that the Rz value was reduced by laser irradiation until it was similar to the as-sprayed Rz_x values reported in Figure 5d.

### 3.3. Second Experimental Series

**Figure 7:**
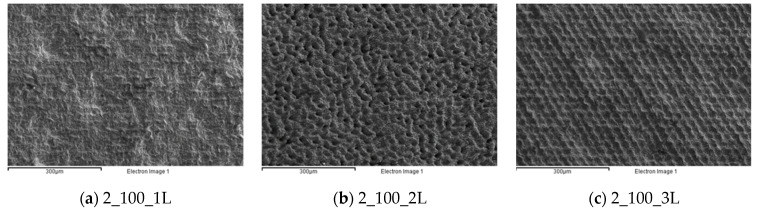

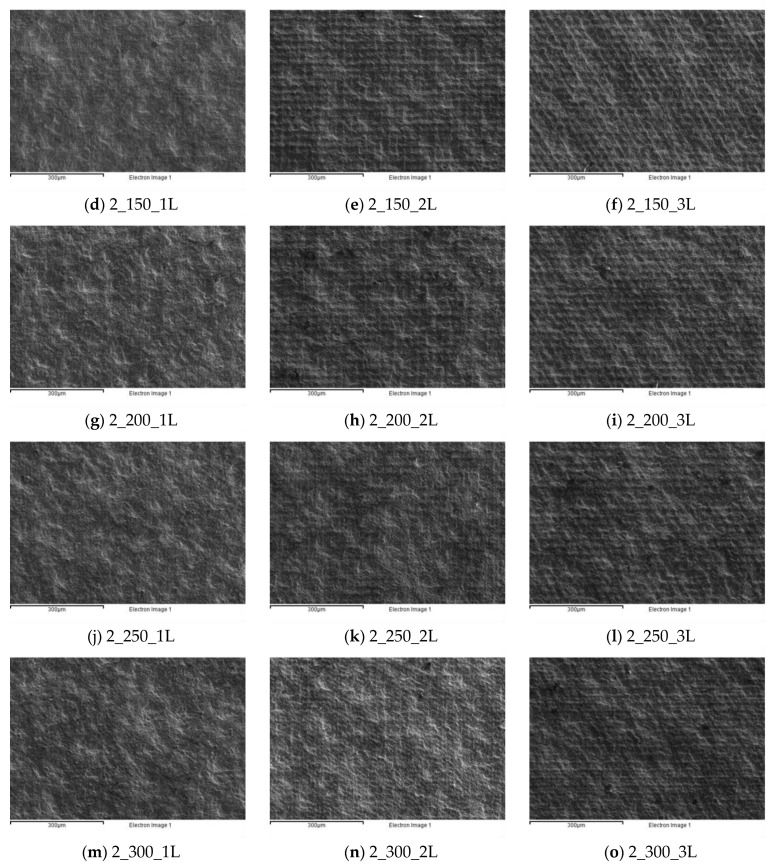
Morphological analysis for the second experimental series. (**a**) 2_100_1L; (**b**) 2_100_2L; (**c**) 2_100_3L; (**d**) 2_150_1L; (**e**) 2_150_2L; (**f**) 2_150_3L; (**g**) 2_200_1L; (**h**) 2_200_2L; (**i**) 2_200_3L; (**j**) 2_250_1L; (**k**) 2_250_2L; (**l**) 2_250_3L; (**m**) 2_300_1L; (**n**) 2_300_2L; (**o**) 2_300_3L.

The qualitative and quantitative analyses of the second experimental series are reported in Figure 7; the coding methods used are coherent with those of the first experimental series. The analyses highlight how the laser beam affected the surface, creating tailored surfaces on the substrate. The one-line strategy was effective up the scanning rate of 200 mm/s (Figure 7g), where it is possible to observe the laser track on the shot peened surface; after that, the starting morphology is predominant (see Figure 7j,m). For the two-line strategy, its effect was to totally change the coating morphology when the scanning rate was less than 200 mm/s (Figure 7b,e,h). For the higher scanning rates, the track effect was still present, but it is possible to observe the original coating surface as well. The results for the three-line strategy were coherent with those of the two-line strategy.

**Figure 8:**
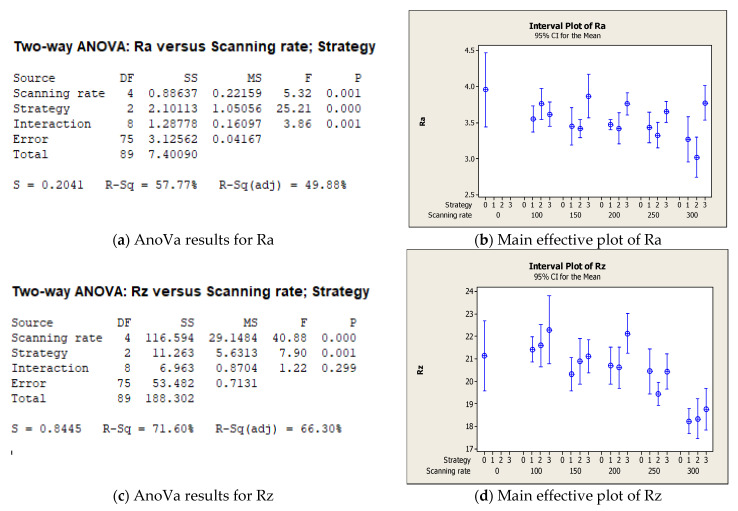
Main results of statistical analysis for the second experimental series. (**a**) AnoVa results for Ra; (**b**) Main effective plot of Ra; (**c**) AnoVa results for Rz; (**d**) Main effective plot of Rz.

Figure 8 reports the main results of the statistical analysis. In this experimental series, the AnoVa demonstrated that the tested process parameters had significant effects on both Ra (Figure 8a) and Rz (Figure 8c). In particular, comparing the results with the initial value (test 0, reported on the left in Figure 8b,d), it is possible to observe a decreasing trend for both values and a significant effect of the two-line strategy, which achieved the highest level of Ra and Rz reduction for all of the tested scanning rates. Moreover, it is possible to observe that the three-line strategy reached the lowest reduction and highest data dispersion.

### 3.4. Third Experimental Series

**Figure 9:**
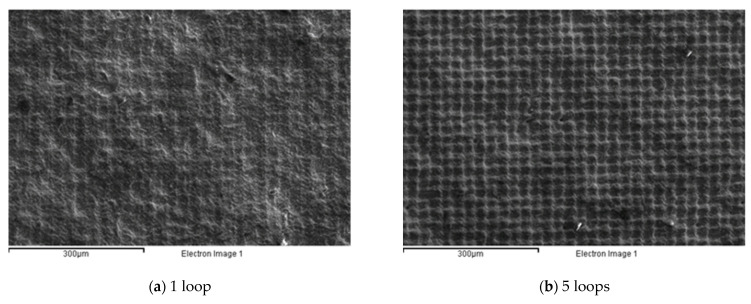

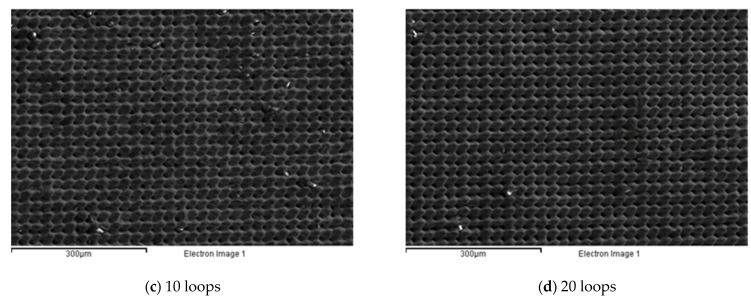
Morphological analysis for the third experimental series. (**a**) 1 loop; (**b**) 5 loops; (**c**) 10 loops; (**d**) 20 loops.

The object of this series was to investigate the effect of the number of loop cycles on the coated surface, mediated by the effect of laser irradiation. Figure 9 presents the effect of the number of loop cycles on the realized morphology. As can be seen from the figure, an increase in the number of loop cycles turned the effect of the laser machining from a soft interaction, which induced a tailored surface (see Figure 9a,b), to a texturing process. In particular, in Figure 9c,d, it is possible to observe how the coating morphology was significantly changed by creating waviness in the surface along the x and y directions.

**Figure 10:**
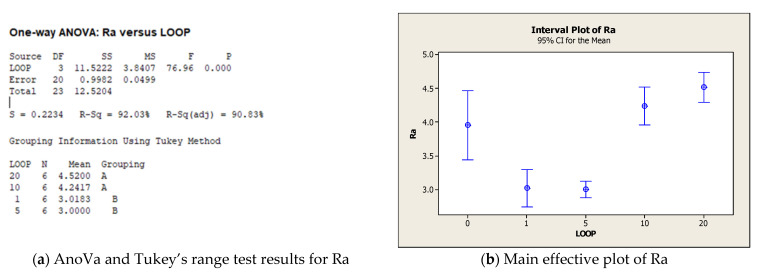

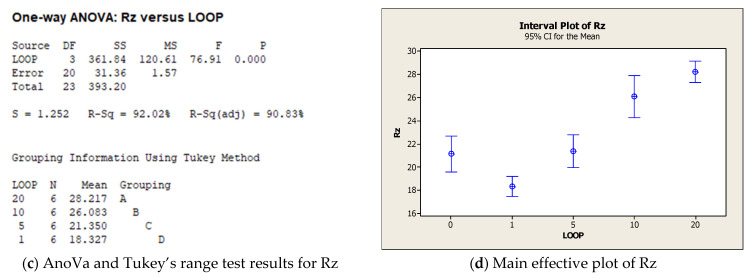
Main results of statistical analysis for the third experimental series. (**a**) AnoVa and Tukey’s range test results for Ra; (**b**) Main effective plot of Ra; (**c**) AnoVa and Tukey’s range test results for Rz; (**d**) Main effective plot of Rz.

The statistical analysis reported in Figure 10 confirmed the results obtained from the quantitative analysis. The AnoVa test reported a significant effect of the number of loop cycles on Ra and Rz. Regarding the Ra analysis, it is possible to observe from Figure 10a that Tukey’s range test identified two main groups (A and B) that demonstrate a significant difference between laser irradiation executed with 1 and 5 loop cycles, compared to 10 and 20 cycles. Moreover, the Ra interval plot highlights how the roughness decreased with a low number of loops, after which it returned to values comparable to the initial one (test 0). In all configurations tested, the standard deviation was lower than that obtained in test 0. Similar results were achieved in the Rz analysis in terms of an increasing trend observed as a function of the number of loop cycles; however, as reported in Figure 10d, with an increase in the number of loop cycles, Rz achieved values higher those in the initial configuration (test 0). Tukey’s range test demonstrated that all loop levels tested were significantly different from each other.

**Figure 11:**
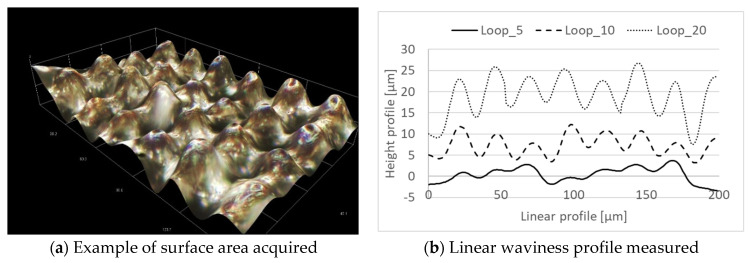
Profile analysis for the third experimental series. (**a**) Example of surface area acquired; (**b**) Linear waviness profile measured.

To better understand the surface texturing observed in Figure 9c,d, coupled with the increased Rz measured in the statistical analysis (Figure 9d), a profile analysis was further executed. In Figure 11a, it is possible to observe an example of the area acquired (200 × 100 µm) for each loop level while, in Figure 11b, the linear profiles extrapolated from the acquisitions are reported as a function of the number of loop cycles. The height profile values were voluntarily increased by 5 and 10 µm, respectively, in the loop 10 and loop 20 tests in order to avoid overlapping graphs, thereby providing better visualization of each single profile. The profiles reported in Figure 11b confirm that homogenous textured surfaces were produced by imposing loop cycles equal to 10 and 20, and further confirms the observed increase in Rz.

## 4. Discussion

**Figure 12:**
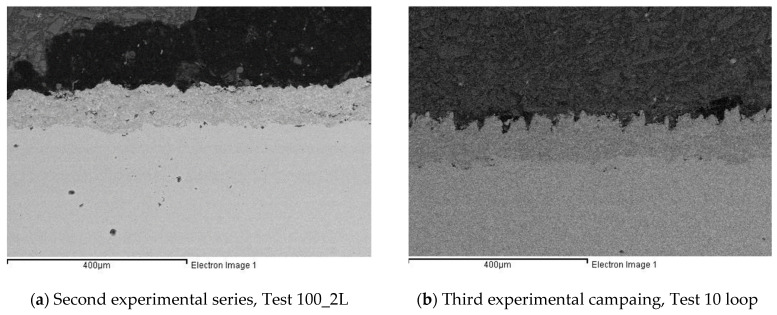
Sample cross section referring to laser polishing (**a**) and texturing (**b**) processes.

The results of this study, regarding the effects of laser irradiation on Cr_3_C_2_-25(Ni20Cr) coatings deposited using an HVOF technique, are summarized below:

- In all experimental series, the qualitative analysis showed an absence of coating damage, such as cracks. Similar results could be found in the cross section reported as an example in Figure 12 in the case of the polishing (Figure 12a) or texturing process (Figure 12b).
- The first experimental series highlighted that the as-sprayed HVOF coatings were characterized by different roughnesses along the x and y directions. The two- and three-line strategies affected the surface, creating a pattern on the coating for all levels of laser scanning rate tested. From a quantitative point of view, the main effect of the tested process parameters was a reduction of Rz along the y direction. This reduction led the sample to have the same Rz values along both directions, thus creating a more homogenous surface.
- The second experimental series showed how, for the surfaces characterized, laser irradiation of a smoother surface results in improved surface properties, allowing for the realization of both a tailored surface and a reduction in terms of roughness; in particular, the use of the two-line strategy with a laser scanning rate equal to 200 mm/s could provide an interesting solution to improving coating performance while inducing an oriented pattern. These results could be useful to improve properties such as wear by means of a reduction of the friction coefficient [28] or corrosion resistance by means of an oxide film on the polished surface [26].
- The third experimental series demonstrated that, by increasing the number of loop cycles, it is possible to improve the average surface roughness (Ra) when the number of loop cycles is equal to 5. When the number of loop cycles increases, the main effect of laser irradiation on the coated surface is to generate a texture characterized by an average roughness comparable to the initial one, but with higher values of Rz. These latter results could be useful for increasing wear behavior or lubrication ability, as has been demonstrated in other studies [21,27].

## 5. Conclusions

In this research, the interactions between Nd:YVO_4_ laser irradiation and Cr_3_C_2_-25 (Ni20Cr) coatings deposited using an HVOF technique were investigated. The effects of laser scanning rate, scan strategy, and number of loop cycles were studied in samples (both as-sprayed and after shot peening). The results highlight the critical issue of realizing laser irradiation on the as-sprayed sample: due to the high starting surface roughness, the main benefits were realizing a more homogenous surface (in terms of Rz) while avoiding the effect of direction. To the contrary, in the case of shot peening surfaces, laser machining led to a polishing process, and it was possible to achieve a significant roughness reduction by imposing a two-line strategy with a laser scanning rate higher than 200 mm/s. Regarding the number of loop cycles, we demonstrated an increased polishing effect when the number of loop cycles was equal to 5, while a texturing process was observed when the number of loop cycles was equal to 10 or 20.

## Figures and Tables

**Table 1:** Fixed laser parameters.

Fixed Laser Process Parameter	Value
Laser Spot Diameter [mm]	0.1
Focal distance [mm]	160
Filling line gap between adjacent laser scan [mm]	0.025
Overlapping track [%]	75%
Laser power [W]	2.2
Laser power density [W/mm^2^]	280
Shielding gas	Air
Irradiated area [mm^2^]	7.5 × 5

**Table 2:** Variable laser process parameters.

Series	Surface Morphology	Scanning Rate [mm/s]	Strategy	Loops
1	As-sprayed	100/150/200/250/300	1/2/3	1
2	Shot peening	100/150/200/250/300	1/2/3	1
3	Shot peening	300	2	1/5/10/20

**Table 3:** Ra and Rz values after HVOF coating with and without shot peening.

Sample	Direction	Ra [µm]	Rz [µm]
As-sprayed	X	5.41 ± 0.15	40.24 ± 1.56
	Y	6.91 ± 0.43	62.88 ± 3.60
After shot peening	X/Y	3.96 ± 0.49	21.13 ± 1.49

## Data Availability

Data available on request due to privacy restrictions.

## References

[1] Brown M.S., Arnold C.B. (2010). Fundamentals of Laser-Material Interaction and Application to Multiscale Surface Modification. Springer Ser. Mater. Sci..

[2] Kusinski J., Kac S., Kopia A., Radziszewska A., Rozmus-Górnikowska M., Major B., Major L., Marczak J., Lisiecki A. (2012). Laser modification of the materials surface layer-a review paper. Bull. Pol. Acad. Sci. Tech. Sci..

[3] Kurella A., Dahotre N.B. (2005). Review paper: Surface modification for bioimplants: The role of laser surface engineering. J. Biomater. Appl..

[4] Tian Y.S., Chen C.Z., Li S.T., Huo Q.H. (2005). Research progress on laser surface modification of titanium alloys. Appl. Surf. Sci..

[5] Giorleo L., Ceretti E., Giardini C. (2016). Optimization of laser micromachining process for biomedical device fabrication. Int. J. Adv. Manuf. Technol..

[6] Krishnan A., Fang F. (2019). Review on mechanism and process of surface polishing using lasers. Front. Mech. Eng..

[7] Ermergen T., Taylan F. (2021). Review on Surface Quality Improvement of Additively Manufactured Metals by Laser Polishing. Arab. J. Sci. Eng..

[8] Etsion I. (2005). State of the art in laser surface texturing. J. Tribol..

[9] Kovalchenko A., Ajayi O., Erdemir A., Fenske G., Etsion I. (2005). The effect of laser surface texturing on transitions in lubrication regimes during unidirectional sliding contact. Tribol. Int..

[10] Etsion I. (2004). Improving tribological performance of mechanical components by laser surface texturing. Tribol. Lett..

[11] Ryk G., Kligerman Y., Etsion I. (2002). Experimental investigation of laser surface texturing for reciprocating automotive components. Tribol. Trans..

[12] Voevodin A.A., Zabinski J.S. (2006). Laser surface texturing for adaptive solid lubrication. Wear.

[13] Vilhena L.M., Sedlaček M., Podgornik B., Vižintin J., Babnik A., Možina J. (2009). Surface texturing by pulsed Nd:YAG laser. Tribol. Int..

[14] Giorleo L., Ceretti E., Giardini C. (2011). ALD coated tools in micro drilling of Ti sheet. CIRP Ann. Manuf. Technol..

[15] Hawthorne H.M., Arsenault B., Immarigeon J.P., Legoux J.G., Parameswaran V.R. (1999). Comparison of slurry and dry erosion behaviour of some HVOF thermal sprayed coatings. Wear.

[16] Picas J.A., Forn A., Matthäus G. (2006). HVOF coatings as an alternative to hard chrome for pistons and valves. Wear.

[17] Lima C.R.C., Guilemany J.M. (2007). Adhesion improvements of Thermal Barrier Coatings with HVOF thermally sprayed bond coats. Surf. Coat. Technol..

[18] Wang Q.-Y., Bai S.-L., Zhao Y.-H., Liu Z.-D. (2014). Effect of mechanical polishing on corrosion behavior of Hastelloy C22 coating prepared by high power diode laser cladding. Appl. Surf. Sci..

[19] Giorleo L., Ceretti E., Montesano L., La Vecchia G.M. (2017). Nd:YOV_4_ laser polishing on WC-Co HVOF coating. AIP Conf. Proc..

[20] Prieske M., Vollertsen F. (2020). Picosecond-laser polishing of CVD-diamond coatings without graphite formation. Mater. Today Proc..

[21] Giorleo L., Montesano L., La Vecchia G.M. (2021). Laser Surface Texturing to Realize Micro-grids on DLC Coating: Effect of Marking Speed, Power, and Loop Cycle. Int. J. Precis. Eng. Manuf..

[22] Tailor S., Vashishtha N., Modi A., Modi S.C. (2019). Structural and mechanical properties of HVOF sprayed Cr_3_C_2_-25%NiCr coating and subsequent erosion wear resistance. Mater. Res. Express.

[23] Da Cunha C.A., Correa O.V., Sayeg I.J., Ramanathan L.V. (2017). High temperature erosion-oxidation resistance of thermally sprayed nanostructured Cr_3_C_2_-25(Ni-20Cr) coatings. Mater. Res..

[24] Morimoto J., Sasaki Y., Fukuhara S., Abe N., Tukamoto M. (2006). Surface modification of Cr_3_C_2_-NiCr cermet coatings by direct diode laser. Vacuum.

[25] Sun G., Tong Z., Fang X., Liu X., Ni Z., Zhang W. (2016). Effect of scanning speeds on microstructure and wear behaviour of laser-processed NiCr-Cr_3_C_2_-MoS_2_-CeO_2_ on 38CrMoAl steel. Opt. Laser Technol..

[26] Scendo M., Zorawski W., Staszewska-Samson K., Goral A. (2021). Influence of laser treatment on the corrosion resistance of Cr_3_C_2_-25(Ni20Cr) cermet coating. Materials.

[27] Góral A., Żórawski W., Lityńska-Dobrzyńska L., Makrenek M., Goły M., Trelka A., Szlezynger M. (2021). Laser modification of the microstructure and mechanical properties of (Cr_3_C_2_-25(Ni20Cr))-5(Ni25C) cermet coatings containing a solid lubricant. Surf. Coat. Technol..

[28] Zhang S.H., Yoon J.H., Li M.X., Cho T.Y., Joo Y.K., Cho J.Y. (2010). Influence of CO_2_ laser heat treatment on surface properties, electrochemical and tribological performance of HVOF sprayed WC-24%Cr_3_C_2_-6%Ni coating. Mater. Chem. Phys..

